# A culturally congruent approach to trauma symptoms detection in first-episode psychosis

**DOI:** 10.4102/sajpsychiatry.v30i0.2260

**Published:** 2024-09-30

**Authors:** Vuyokazi Ntlantsana, Usha Chhagan, Enver Karim, Saeeda Paruk, Andrew Tomita, Bonginkosi Chiliza

**Affiliations:** 1 Discipline of Psychiatry, School of Clinical Medicine, University of KwaZulu-Natal, Durban, South Africa; 2 KwaZulu-Natal Research Innovation and Sequencing Platform (KRISP), College of Health Sciences, University of KwaZulu-Natal, Durban, South Africa; 3 Centre for Rural Health, School of Nursing and Public Health, University of KwaZulu-Natal, Durban, South Africa

## Background

Psychosis is a group of symptoms indicating serious mental illness that burdens society with healthcare costs, caregiver responsibility and increased years lived with disability.^1^ The association between childhood trauma, lifecourse trauma and post-traumatic stress disorder (PTSD) sequela has been established in populations with psychosis.^2,3,4^ Childhood trauma is a shared risk factor for the development of psychosis and PTSD.^2^ There are high rates of PTSD in people with early psychosis and promising results for psychological interventions for affected individuals, with improvement of both PTSD and psychosis symptoms.^5,6^ However, in clinical practice, trauma symptoms are often overlooked, and PTSD diagnosis is even more likely to be missed in patients who present with psychotic disorders.^7^

Post-traumatic stress disorder often presents a diagnostic dilemma in patients with psychosis, as symptoms may be difficult to distinguish,^8^ with flashbacks possibly being difficult to differentiate from hallucinations of psychosis, for example. Furthermore, culture influences the presentation of psychopathology, leading to potentially difficult encounters between the healthcare provider and patient.^9,10^ On this premise, patients with psychosis presenting with cultural symptoms of distress are even more likely to be missed and therefore sub-optimally managed. This study therefore aimed to assess the prevalence of childhood trauma, lifecourse trauma and PTSD in patients with first episode psychosis (FEP) in South Africa using a culturally appropriate approach.

## Methods

The letter describes baseline data of a prospective cohort study of patients with FEP, which was collected between April 2019 and December 2022, having been described by Chhagan, Ntlantsana.^11^ Briefly, in- and out-patients from five eThekwini District Hospitals in KwaZulu-Natal (KZN) Province were included, specifically those aged 18–45 years who had a minimum education level of Grade 7, a confirmed diagnosis of one of the schizophrenia spectrum disorders (according to DSM-5), received less than 6 weeks of antipsychotics and did not have another serious medical condition. The Mini International Neuropsychiatric Interview (MINI) schedule for psychosis was used to confirm the diagnosis,^12^ and the Childhood Trauma Questionnaire (CTQ) was used to assess for five domains of childhood trauma, namely emotional, physical and sexual abuse, and emotional and physical neglect.^13^ Participants meeting threshold for moderate or severe exposure in each CTQ domain were classified as positive, while those with negative or mild scores were categorised as negative, as observed in a prior study of participants with psychotic symptoms.^14^ The Positive and Negative Symptoms Scale (PANSS) assessed for severity of psychosis symptoms.^15^ The PTSD assessments were conducted 3 months after enrolment in the study. The PTSD Checklist for DSM-5 (PCL-5) with Life Events Checklist for DSM-5 (LEC-5) was used to assess exposure to lifetime traumatic events and to screen for PTSD.^17^ Additionally, the Zulu Culture-specific Trauma Experience Questionnaire (Z-CTEQ) was used to screen for trauma experiences and PTSD related specifically to Zulu cultural experiences.^16,17^ Data were collected by qualified medical doctors or psychiatrists, and a trained research assistant with a tertiary education qualification in psychology assisted with data collection for self-report tools.

### Ethical considerations

The study was approved by the University of KwaZulu-Natal Biomedical Research Ethics Committee (No. BCA 571/18). Written consent was provided by all participants, with the anonymised participant records being kept in a locked office. Data were entered into a REDCap database, a secure research online database.^18^

## Results

Sixty-four participants were included in the study, their median age being 24 years (interquartile range [IQR]: 20–30), of whom 42 (66%) were male, 11 (17%) had attained a tertiary level education, 43 (67%) were unemployed, 10 (16%) were employed and 11 (17%) were students. Thirty-nine (61%) reported at least one lifetime trauma event, according to the LEC-5, and the most prevalent events reported were physical assault (61%), a life-threatening illness (46%), natural hazard (42%), transportation accident (42%), and learning about or witnessing a sudden violent death (39%). The number of participants who reached the threshold for one or more childhood trauma domains was 41 (64%). A comparative analysis of psychosis and childhood trauma by domains is presented in Table 1, with 16 participants (25%) scoring positive for PTSD, according to the PCL-5, and an additional three (5%) for PTSD, according to the Z-CTEQ. Physical abuse in childhood and lifecourse physical assault were significantly associated with PTSD, (odds ratio [OR]: 5.1, *p* = 0007 and OR 6.1, *p* = 0.028, respectively), while sex and HIV status were not significantly associated with PTSD, (OR 1.1, *p* = 0.882 and OR 0.5, *p* = 0.326).

**TABLE 1:** Clinical symptoms and childhood trauma by sex.

Variable	Female (*N* = 22)		Male (*N* = 42)		*p*	Test
	*n*	%	*n*	%		
**Psychosis symptom scores (PANSS)**						
Total score, median	80.4	19.3	79.6	19.7	0.88	Two sample t test
Negative, median	19.0	14.0, 23.0	18.5	10.0, 33.0	0.96	Wilcoxon rank-sum
Disorganised, mean	16.9	6.5	17.3	5.4	0.78	Two sample t test
Positive, mean	8.5	5.0, 15.0	8.5	6.0, 13.0	0.94	Wilcoxon rank-sum
Excited, mean	13.0	9.0, 14.0	7.0	5.0, 11.0	< 0.001	Wilcoxon rank-sum
Anxiety scores, median	6.0	5.0, 7.0	4.5	3.0, 5.0	0.003	Wilcoxon rank-sum
**Childhood trauma scores**						
Emotional abuse	-	-	-	-	0.002	Pearson’s chi-squared
Negative	8	36.4	32	76.2	-	-
Positive	14	63.6	10	23.8	-	-
Physical abuse	-	-	-	-	0.13	Pearson’s chi-squared
Negative	11	50.0	29	69.0	-	-
Positive	11	50.0	13	31.0	-	-
Sexual abuse	-	-	-	-	0.017	Pearson’s chi-squared
Negative	6	40.0	27	75.0	-	-
Positive	9	60.0	9	25.0	-	-
Emotional neglect	-	-	-	-	0.21	Pearson’s chi-squared
Negative	16	72.7	36	85.7	-	-
Positive	6	27.3	6	14.3	-	-
Physical neglect	-	-	-	-	0.38	Pearson’s chi-squared
Negative	9	40.9	22	52.4	-	-
Positive	13	59.1	20	47.6	-	-

## Discussion

The study determined the prevalence of lifecourse and childhood trauma as well as PTSD in a cohort of patients with FEP, with lifecourse and childhood trauma being highly prevalent, highlighting the importance of enquiring about trauma as part of their clinical assessment. The PTSD was detected in almost a third of participants, indicating that it is highly comorbid with FEP. The use of a culturally congruent assessment tool increased the detection of PTSD.

At 41%, the prevalence of any childhood trauma was reported at a higher rate than that found by Kilian et al.^19^ in their cohort of patients with first-episode schizophrenia in the Western Cape Province (South Africa). Lifecourse trauma was detected in a third of participants in our study, a considerably lower proportion compared to that reported elsewhere in South Africa, with trauma exposure rates having been reported to be up to 92% in populations with psychosis.^20^ In their multi-site study in South Africa, Stevenson et al.^20^ found that trauma rates were significantly higher in people with psychotic and related disorders compared to control groups. There are various possible reasons for lower rates of trauma in our cohort, one being that our cohort were younger than the participants in other studies, thus increasing the likelihood of exposure to more lifetime trauma events. Another possibility may be related to under reporting, as was found in a study by Burns, Coughlan and Cannon,^21^ where 16% of participants in a community sample of adolescents did not report trauma experiences, this being significantly associated with a history of psychotic experiences.

Physical abuse in childhood and a history of physical assault were associated with higher odds of screening positive for PTSD, this being consistent with studies that reported that intentional trauma increases the likelihood of developing PTSD over time.^22,23^ While PTSD rates are known to be high in populations with psychosis, as a comorbid condition, it remains largely undetected in clinical practice, thus negatively impacting on outcomes in this group of patients.^7^ In their systematic review examining undetected PTSD in secondary mental health services, Zammit et al.^7^ found 28% of patients with psychosis had PTSD on screening, but no documentation of the condition in their clinical records, suggesting a missed diagnosis. With almost a third of participants screening positive for PTSD in the review, the prevalence is consistent with our findings.

Culture influences the development of psychopathology in many populations globally and is central in the mental health literacy of many African people in KwaZulu-Natal Province, where health is understood as a harmonious relationship with their ancestors.^10,24,25,26,27^ Consistent with findings by Madigoe et al.,^16^ we increased the detection of PTSD with the use of a culturally congruent assessment tool that inquired about potentially traumatic cultural experiences, such as spiritual events, which are historically not part of traditional methods of assessments. Although the additional 5% PTSD detected using the culturally congruent tool may be interpreted as modest, it is important to acknowledge that these individuals had a comorbid diagnosis that would have otherwise gone undetected. Furthermore, Madigoe et al.^16^ detected rates of up to 19% in their sample. The inclusion of indigenous knowledge systems in assessments enhances relevance and responsiveness to local symptoms of distress.^28^

We notice that the prevalence rates and associations in our study need to be interpreted with caution because of the small sample size. However, the study brings to the forefront two important factors; firstly, mental healthcare providers treating populations with early psychosis need to explore the presence of and possible impact of childhood and lifecourse trauma, as well as the possibility of comorbid PTSD. Secondly, practitioners must consider the role of culture in clinical presentation and management of mental health.^10^ We make a case for the development and inclusion of culturally congruent assessments and interventions to be included as part of comprehensive mental healthcare package.
