# Corrigendum: A culturally congruent approach to trauma symptom evaluation improves detection of PTSD in people with a first-episode of psychosis in South Africa

**DOI:** 10.4102/sajpsychiatry.v30i0.2406

**Published:** 2024-10-31

**Authors:** Vuyokazi Ntlantsana, Usha Chhagan, Enver Karim, Saeeda Paruk, Andrew Tomita, Bonginkosi Chiliza

**Affiliations:** 1 Discipline of Psychiatry, School of Clinical Medicine, University of KwaZulu-Natal, Durban, South Africa; 2 KwaZulu-Natal Research Innovation and Sequencing Platform (KRISP), College of Health Sciences, University of KwaZulu-Natal, Durban, South Africa; 3 Centre for Rural Health, School of Nursing and Public Health, University of KwaZulu-Natal, Durban, South Africa

This corrigendum addresses errors that resulted in missing or misleading text in an otherwise reliable publication, Ntlantsana V, Chhagan U, Karim E, Paruk S, Tomita A, Chiliza B. A culturally congruent approach to trauma symptoms detection in first-episode psychosis. S Afr J Psychiatry. 2024;30(0), a2260. https://doi.org/10.4102/sajpsychiatry.v30i0.2260.

The original incorrect wording on page 2, in the second paragraph of the ‘Discussion’ section, read:

At 41%, the prevalence of any childhood trauma was reported at a higher rate than that found by Kilian et al.^19^ in their cohort of patients with first-episode schizophrenia in the Western Cape Province (South Africa).

The revised and updated wording should read:

At 64%, the prevalence of any childhood trauma was reported at a higher rate than that found by Kilian et al.^19^ in their cohort of patients with first-episode schizophrenia in the Western Cape Province (South Africa).

There was also an intext error on page 3, where Madigoe et al. was cited as detecting rates of up to 19% in their sample. The correct rate is 10%.

The original incorrect wording on page 3, read:

Furthermore, Madigoe et al.^16^ detected rates of up to 19% in their sample.

The revised and updated wording should read:

Furthermore, Madigoe et al.^16^ detected rates of up to 10% in their sample.

The authors apologise for this error. The correction does not change the study’s findings, its significance or overall interpretation of the study’s results or the scientific conclusions of the article in any way.

